# Comparison of Immune Checkpoint Inhibitor (ICI) Myocarditis and Non-ICI Myocarditis Using Cardiovascular Magnetic Resonance: A Single-Centre Retrospective Observational Study

**DOI:** 10.3390/jcm14217809

**Published:** 2025-11-03

**Authors:** Ella Jacobs, Anthony Yip, Alison Hodge, Denise McLean, Joon Lee, Victoria Parish, Susan Ellery, Anna Olsson-Brown, Alexander Liu

**Affiliations:** 1 Sussex Cardiac Centre, Royal Sussex County Hospital, Brighton BN2 5BE, UK; ella.jacobs@nhs.net (E.J.); anthony.yip2@nhs.net (A.Y.); victoria.parish@nhs.net (V.P.); susan.ellery1@nhs.net (S.E.); 2 Sussex Cancer Centre, Royal Sussex County Hospital, Brighton BN2 5BE, UK; alison.hodge3@nhs.net (A.H.); denise.mclean2@nhs.net (D.M.); a.olsson-brown@nhs.net (A.O.-B.); 3 Department of Radiology, Royal Sussex County Hospital, Brighton BN2 5BE, UK; joon.lee@nhs.net

**Keywords:** immune checkpoint inhibitor, myocarditis, myocardial oedema, T1-mapping, T2-mapping, extracellular volume fraction, LV dysfunction

## Abstract

**Background**: Differentiating between immune checkpoint inhibitor (ICI) myocarditis and non-ICI myocarditis is clinically important. Cardiovascular magnetic resonance (CMR) is a well-established method for diagnosing acute myocarditis. The value of CMR for distinguishing ICI myocarditis from non-ICI myocarditis remains unclear, which this study sought to determine. **Methods**: A total of 54 patients (*n* = 26 ICI myocarditis; *n* = 28 non-ICI myocarditis) underwent clinical CMR for the assessment of cardiac function (cines), myocardial fibrosis (native T1-mapping, extracellular volume [ECV] fraction, late gadolinium enhancement [LGE]) and myocardial oedema (native T2-mapping). **Results**: ICI myocarditis patients were older than non-ICI myocarditis patients (75 years [71–78] vs. 39 years [30–64]; *p* < 0.001). Both groups had similar left ventricular (LV) ejection fraction (58 ± 11% vs. 58 ± 6%; *p* = 0.970). ICI myocarditis and non-ICI myocarditis patients also had similar native myocardial T1 values (1041 ± 84 ms vs. 1063 ± 60 ms; *p* = 0.281), native myocardial T2 values (59 ± 6 ms vs. 59 ± 6 ms; *p* = 0.943) and ECV (0.32 ± 0.07 vs. 0.31 ± 0.04; *p* = 0.403). Native myocardial T1 values (Rho = −0.553) and ECV (Rho = −0.502) were significantly associated with LVEF in non-ICI myocarditis patients (both *p* < 0.05). There was no significant association between myocardial T1 values, T2 values or ECV, with LVEF, in ICI myocarditis patients (all *p* < 0.05). Non-ICI myocarditis patients had a greater frequency of LGE in the LV compared to ICI myocarditis patients (89% vs. 52% *p* = 0.005). However, the pattern of LGE was similar between the two patient groups (mostly subepicardial and/or mid-wall). **Conclusions**: In this single centre retrospective cohort, the findings suggest that quantitative parametric mapping methods by CMR may not differentiate between ICI vs. non-ICI myocarditis. Further work is needed to assess the value of CMR for diagnosing standalone ICI myocarditis.

## 1. Introduction

Myocarditis is an inflammatory condition which can lead to cardiac contractile dysfunction and heart failure [1,2,3]. Viral myocarditis is one of the commonest aetiologies, involving pathogens such as adenoviruses, enteroviruses, parvovirus B19 and influenza [1,4]. More recently, the increasing use of novel immune checkpoint inhibitor (ICI) therapies for the treatment of a wide range of cancers is paralleled by an increasing incidence of ICI myocarditis [5,6,7]. ICIs, including agents such as ipilimumab (anti–CTLA-4), nivolumab and pembrolizumab (anti–PD-1), and atezolizumab (anti–PD-L), have revolutionised the management of patients with cancer, by targeting immune checkpoint inhibitory pathways including CTLA-4, PD-L and PD-L1 [5,8,9].

ICI myocarditis is associated with a high risk of morbidity and mortality [5]. Early recognition can enable urgent initiation of immunosuppressive therapies and potentially life-saving treatments [10]. ICI myocarditis can have a similar presentation to other forms of acute myocarditis, and it is not always easy to distinguish between ICI vs. non-ICI myocarditis clinically [10]. However, clinical management is often very different between these two types of conditions [5]. Whilst ICI myocarditis may require immunosuppressive therapies such as corticosteroids, and possibly withholding of the offending immunomodulatory drug [5,6,7], non-ICI myocarditis may be managed conservatively with supportive treatments only [5]. Therefore, being able to distinguish between ICI vs. non-ICI myocarditis is important from both diagnostic and therapeutic standpoints.

Cardiovascular magnetic resonance (CMR) is a multi-parametric and non-invasive imaging modality which plays a central role in the diagnosis of myocarditis [11,12]. CMR offers a one-stop evaluation of cardiac volumes, systolic function and tissue characterisation [11,12,13,14]. Quantitative T1 and T2 mapping techniques have enabled the detection of myocardial fibrosis and oedema [12]. Late gadolinium enhancement (LGE) imaging is a valuable tool for assessing the patterns of myocardial fibrosis in myocarditis [15,16].

CMR is commonly used to assess patients with acute myocarditis, its ability to distinguish between ICI and non-ICI myocarditis. Recent studies suggest that ICI myocarditis may exhibit distinctive imaging phenotypes, with lower LGE prevalence and less extensive myocardial oedema compared to non-ICI myocarditis [10]. There remains limited head-to-head comparison data between ICI and non-ICI myocarditis using CMR for differentiation. Further, the effect of pathologies such as myocardial oedema and fibrosis on left ventricular (LV) systolic function in ICI vs. non-ICI myocarditis is unclear. These are important knowledge gaps to fulfil, given the contrasting management strategies between the two types of conditions.

This study sought to assess the ability of CMR to distinguish between ICI vs. non-ICI myocarditis. The primary objective was to assess the between-group differences (ICI vs. non-ICI myocarditis) in myocardial oedema and fibrosis as assessed using CMR techniques such as parametric mapping methods and LGE. The secondary objective was to assess the effect of myocardial oedema and fibrosis on LV ejection fraction (LVEF) in these disease conditions.

## 2. Methods

### 2.1. Study Subjects

In this retrospective, single-centred observational study, patients (aged 18 or over) with clinically suspected acute myocarditis who underwent CMR scans between August 2023 and August 2025 at the Royal Sussex County Hospital (a UK tertiary cardiac centre) were retrospectively screened.

### 2.2. Inclusion and Exclusion Criteria

In the ICI myocarditis patient cohort, a clinical diagnosis of ICI myocarditis was made by a regional cardio-oncology MDT meeting (consisting of a consensus agreement between consultant cardiologists and oncologists) as patients taking one or more ICI agents who subsequently developed a rise in serum cardiac injury markers (cardiac troponins and/or natriuretic peptides) with evidence of acute myocarditis as reported on a clinical CMR scan by a consultant cardiologist or radiologist. As part of routine care, patients with ICI myocarditis were treated with immunosuppressive therapy by their physicians on clinical grounds, before their CMR scans had taken place.

In the non-ICI myocarditis patient cohort, non-ICI myocarditis was diagnosed in patients who had evidence of myocarditis on clinical CMR (as reported by a consultant cardiologist or radiologist), who were not taking ICI and had an acute rise in serum cardiac injury markers (cardiac troponins and/or natriuretic peptides). In both patient cohorts, obstructive coronary artery disease was excluded as the cause of the changes in cardiac injury markers by either coronary artery imaging, CMR or by consensus at the cardio-oncology MDT meeting.

**Figure 1:**
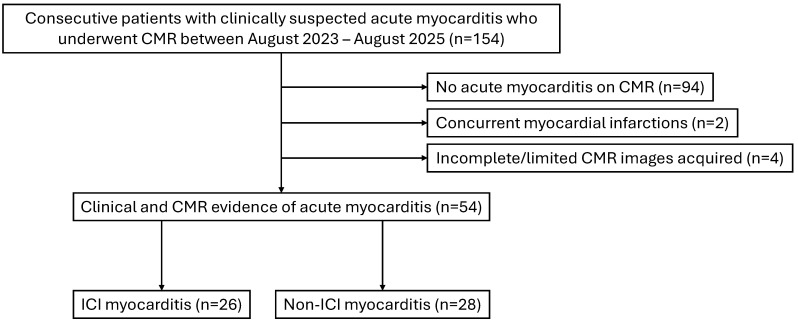
Study flowchart. CMR: cardiovascular magnetic resonance; ICI: immune checkpoint inhibitor.

During the study period, 154 consecutive patients with clinically suspected acute myocarditis who underwent CMR were screened. One hundred patients were excluded due to: (i) no evidence of acute myocarditis on CMR (*n* = 94); (ii) presence of concurrent myocardial infarction (*n* = 2); (iii) incomplete/limited CMR images precluding meaningful analysis (*n* = 4). A total of 54 patients were included in the final analysis (*n* = 26 ICI myocarditis; *n* = 28 non-ICI myocarditis). Figure 1 shows the study flowchart.

### 2.3. Cardiovascular Magnetic Resonance (CMR)

CMR scans were performed at 1.5-Tesla (Ingenia Ambition, Philips Healthcare, Best, The Netherlands) using a clinical protocol as previously described [11,12]. Cine images were acquired in long-axis and short-axis image positions, using a balanced steady-state-free-precession sequence [11]. Native T1-maps were acquired in the mid-ventricular short-axis slice position using a Modified Look-Locker Inversion recovery (MOLLI) sequence with a 5s(3s)3s scheme, as provided by the vendor package. Native T2-mapping was performed using a vendor-provided sequence (GraSE 9 echo) in the same slice positions as the native T1-maps. LGE imaging was performed in long-axis and short-axis image positions, around 7 min after an intravenous bolus injection of a gadolinium-based contrast agent (0.1 mmol/kg; Dotarem, Guerbet, France), which was followed by an intravenous saline flush. Post-contrast T1-mapping was performed in the same slice positions as the native T1-maps, around 15 min after injection of the contrast agent.

### 2.4. CMR Image Analysis

CMR images were analysed using a commercially available software (cvi42, Circle Cardiovascular Imaging, Calgary, AB, Canada). Cardiac volume and systolic function were analysed by clinical CMR consultants. The same clinical CMR consultants also visually assessed the LGE images. Myocardial T1 and T2 values were derived by placing endocardial and epicardial contours on the T1- and T2-maps, avoiding artefacts and the blood–myocardium interface, as previously described [13]. Synthetic extracellular volume fraction (ECV) was derived as previously described [17]. ROIs were placed in the left ventricular (LV) blood pool on both the native and post-contrast T1-maps in order to estimate the T1 values of the LV blood (T1–blood) [17]. Synthetic haematocrit was derived using the previously published formula (922.6 × [1 ÷ T1–blood]) − 0.1668 [17], specific for MOLLI sequences at 1.5-Tesla.

### 2.5. Statistical Analysis

Parametric data were shown as mean ± 1 standard deviation (SD). Non-parametric data were displayed as median [interquartile range]. Comparisons between two groups of parametric data were made using the independent samples Student *t*-test. Comparisons between two groups of non-parametric data were made using the Mann–Whitney U test. Two groups of categorical data were compared using Fisher’s exact test. Correlations between two continuous variables were evaluated using Spearman’s rank correlation coefficient. In case of missing data, the total numbers of data points are indicated in brackets in the Tables. *p* values < 0.05 were considered to be statistically significant. Statistical analysis was performed using a commercially available software (MedCalc, vers. 20.104, Mariakerke, Belgium) and then validated by another observer.

### 2.6. Ethical Approval

The Research and Innovation department of the University Hospitals Sussex NHS Foundation Trust previously approved this retrospective study on 16 December 2024, and informed consent from patients was waived.

## 3. Results

### 3.1. Clinical Characteristics of ICI vs. Non-ICI Myocarditis

Patients with ICI myocarditis were older than patients with non-ICI myocarditis (75 years (71–78) vs. 39 years (30–64); *p* < 0.001; Table 1). Of the cardiac symptoms, patients with ICI myocarditis experienced chest pain less commonly, as compared to patients with non-ICI myocarditis (4% vs. 68% *p* < 0.001; Table 1). The frequency of other cardiac symptoms, such as dyspnoea, palpitations, and syncope/presyncope, was similar between the two patient groups (Table 1). Patients with ICI myocarditis had a similar level of co-morbidities compared to patients with non-ICI myocarditis (Table 1).

Guideline-directed heart failure medical therapy was more commonly administered to patients with ICI myocarditis, as compared to non-ICI myocarditis (Table 1). These included ACE-I/ARB/ARNI, Beta-blocker, MRA and SGLT-2 inhibitor (all *p* < 0.05; Table 1). Other medications, including loop diuretics, antiplatelet drugs, statins, and anticoagulants, were similarly used in both patient groups (Table 1).

Patients with non-ICI myocarditis had significantly higher peak hs-cTnT levels compared to patients with ICI myocarditis (77 ng/L (51–169) vs. 294 ng/L (57–1018); *p* = 0.039; Table 1). However, patients with ICI myocarditis had significantly higher peak NT-pro BNP levels compared to patients with non-ICI myocarditis (7838 ng/L (2412–13,116) vs. 1631 ng/L (612–4147); *p* = 0.015; Table 1).

### 3.2. Characteristics of Patients with ICI Myocarditis

Of the ICI myocarditis patients, the lung was the commonest site for primary malignancy, followed by skin (27%), the tongue (12%) and the bladder (8%; Table 2). Pembrolizumab (31%), Ipilimumab/Nivolumab (19%), Atezolizumab (12%) and Cemiplimab (12%) were amongst the most commonly used ICI agents (Table 2). Most of the ICI patients were treated with immunosuppression, of which the most frequently used regimens included intravenous (IV) methylprednisolone (81%), oral prednisolone (92%), mycophenolate mofetil (58%), tacrolimus (19%), infliximab (19%) and azathioprine (12%; Table 2).

### 3.3. CMR Parameters of Patients

Patients with ICI myocarditis had similar LVEF (58 ± 11% vs. 58 ± 6%, *p* = 0.970) and RVEF (57 ± 6% vs. 59 ± 10%; *p* = 0.341), as compared to patients with non-ICI myocarditis (Table 3). LV mass index was also similar between the two patient groups (52 g/m^2^ (44–57) vs. 58 g/m^2^ (48–67); *p* = 0.051; Table 3).

Patients with non-ICI myocarditis had a higher frequency of LGE presence in the LV compared to those with ICI myocarditis (89% vs. 52%; *p* = 0.005; Table 3). The majority of LGE patterns was limited to subepicardial or mid-wall distributions, with a minority being transmural/subendocardial (Table 3). Subepicardial/mid-wall LGE distribution was more frequently seen in non-ICI myocarditis patients compared to ICI myocarditis patients (41% vs. 0%; *p* < 0.001; Table 3). Other LGE distributions were similar between the two patient groups. No RV LGE was observed in either patient cohort.

Both patients with ICI myocarditis and patients with non-ICI myocarditis had similar native myocardial T1 values (1041 ± 84 ms vs. 1063 ± 60 ms; *p* = 0.281), native myocardial T2 values (59 ± 6 ms vs. 59 ± 6 ms; *p* = 0.943) and synthetic ECV (0.32 ± 0.07 vs. 0.31 ± 0.04; *p* = 0.403; Table 3).

### 3.4. Relationship Between Markers of Oedema and Diffuse Fibrosis and LVEF

**Figure 2:**
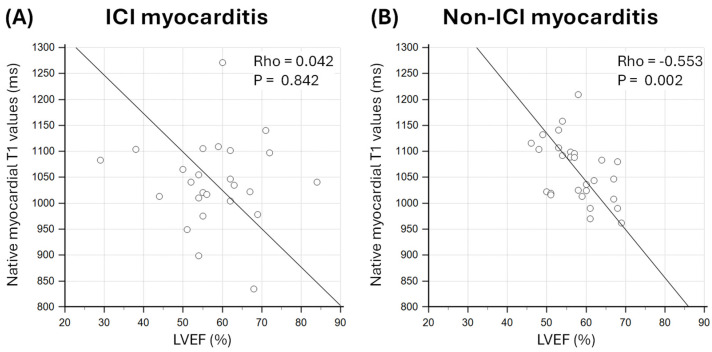
Association between native myocardial T1 values and left ventricular ejection fraction (LVEF) in patients with (**A**) immune checkpoint inhibitor (ICI) myocarditis and (**B**) non-ICI myocarditis.

There was a weak and non-significant negative correlation between native myocardial T1 values and LVEF in patients with ICI myocarditis (Rho = 0.042; *p* = 0.842; Figure 2); however, in patients with non-ICI myocarditis, there was a significant and stronger negative correlation (Rho = −0.553; *p* = 0.002; Figure 2).

**Figure 3:**
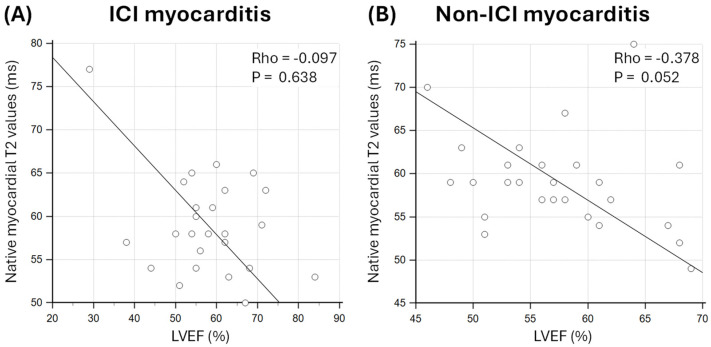
Association between native myocardial T2 values and left ventricular ejection fraction (LVEF) in patients with (**A**) immune checkpoint inhibitor (ICI) myocarditis and (**B**) non-ICI myocarditis.

Native myocardial T2 values were non-significantly correlated with LVEF in both patients with ICI myocarditis and patients with non-ICI myocarditis (Rho = −0.095; *p* = 0.638 and Rho = −0.378; *p* = 0.052, respectively; Figure 3).

**Figure 4:**
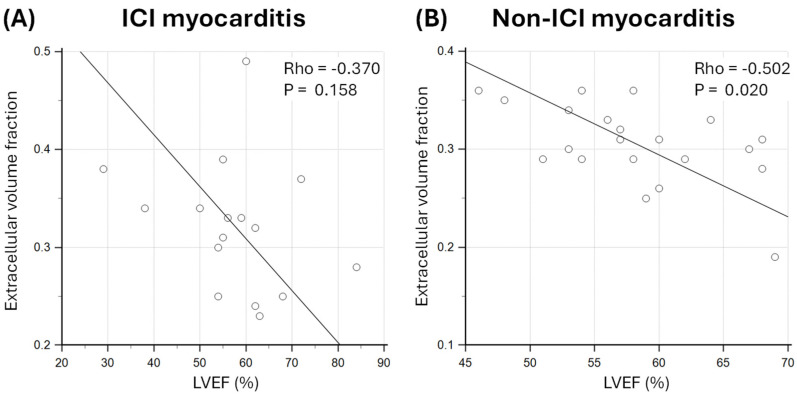
Association between extracellular volume (ECV) fraction and left ventricular ejection fraction (LVEF) in patients with (**A**) immune checkpoint inhibitor (ICI) myocarditis and (**B**) non-ICI myocarditis.

There was a weak and non-statistically significant relationship between ECV and LVEF in ICI myocarditis patients (Rho = −0.370; *p* = 0.158; Figure 4). There was a statistically significant negative correlation between ECV and LVEF in patients with non-ICI myocarditis (Rho = −0.502; *p* = 0.020; Figure 4).

## 4. Discussion

This study assessed the ability of CMR to distinguish between ICI myocarditis and non-ICI myocarditis using pixel-wise native and post-contrast parametric mapping methods. The main findings of this study are: (i) patients with ICI vs. non-ICI myocarditis have similar biventricular systolic function; (ii) a greater frequency of non-ICI myocarditis patients had LGE compared to ICI myocarditis patients; (iii) patients with ICI and non-ICI myocarditis had similar native myocardial T1 and T2 values, as well as ECV; and (iv) there was no significant association between these parametric mapping based parameters with LVEF in ICI myocarditis patients, whilst significant associations were observed between native T1 values (and ECV) with LVEF in non-ICI myocarditis patients.

The findings suggest that differentiating between ICI- vs. non-ICI myocarditis using CMR quantitative parametric mapping techniques alone (T1-/T2-mapping/ECV) may be challenging. Although non-ICI patients had a greater frequency of LGE in the LV, the pattern was non-specific in distinguishing against ICI myocarditis. The association between parametric mapping techniques and LVEF was more pronounced in non-ICI myocarditis patients, as compared to ICI myocarditis patients. This indicates that cardiac contractile function may be more vulnerable to changes in myocardial oedema and fibrosis in non-ICI myocarditis. These findings require further validation in large patient cohorts.

### 4.1. Myocardial Oedema and Inflammation in Myocarditis

Myocardial oedema is a hallmark of a range of inflammatory heart diseases [18,19]. In myocarditis, direct invasion of the cardiomyocyte, along with systemic inflammatory conditions, can lead to myocardial injury [1,2,4,20,21,22,23,24,25,26,27,28]. In ICI myocarditis, it is believed that immune activation leads to myocardial injury, which can be sustained with continued ICI therapy [5]; though the exact pathophysiological mechanism remains incompletely elucidated [29,30,31]. The manifestation of acute myocardial injury is often evident in the CMR images of patients with myocarditis, including elevated native myocardial T1 and T2 values [12]. Elevated ECV has also been reported in myocardial injury in myocarditis, which may also be a reflection of myocardial fibrosis [12,32,33].

To distinguish between ICI and non-ICI myocarditis, there needs to be a numerical difference in the parametric mapping values between the two conditions. However, this study suggests that native myocardial T1 and T2 values (as well as ECV) are similar between the two types of myocarditis. This casts doubt over the sensitivity of the techniques used with respect to the biological differences between the two conditions. In other words, the differences in pathology between ICI and non-ICI myocarditis may be too small to elicit differences in the parametric mapping results. Further, it is expected that both types of myocarditis could have significant overlaps, leading to similar clinical presentations.

Whilst numerical overlaps in myocardial parametric mapping values may prohibit their differentiation, the fact that ICI myocarditis patients underwent CMR after immunosuppressive therapy meant that the degree of myocardial inflammation may have been blunted at the scan. This may mask differences in the CMR results between ICI vs. non-ICI myocarditis patients. Furthermore, ICI myocarditis patients were older than non-ICI patients in this study, which may also affect the CMR results. Future work is required to assess the effect of pre-treatment CMR in ICI myocarditis patients, using an age- or propensity-matched study design.

The results of the study highlight the challenge in distinguishing between the two conditions using CMR alone, which may have significant clinical implications since the two types of myocarditis often require contrasting therapeutic strategies [4,5,6,14,21,22,23,34]. For instance, ICI myocarditis may warrant immunosuppression, whilst non-ICI myocarditis may require only supportive treatment, provided that cardiac function and clinical stability are not significantly compromised [5,22].

### 4.2. Effect of Myocardial Oedema and Fibrosis on LV Function

Notably, patients with non-ICI myocarditis had a pattern of LVEF that was significantly influenced by native myocardial T1 values and ECV. Native myocardial T2 values were also associated with LVEF in non-ICI myocarditis patients, albeit this association did not reach statistical significance. Contrarily, the LVEF of patients with ICI myocarditis was essentially uninfluenced by the level of diffuse myocardial fibrosis (native myocardial T1 values and ECV) and myocardial oedema (native myocardial T2). This contrast in associations is challenging to explain fully, which may reflect, to some extent, differences in the pathogenesis of LV systolic dysfunction in both myocarditis conditions. Further work is needed to elucidate the underlying mechanism of LV dysfunction in ICI myocarditis.

The greater burden of LGE in non-ICI myocarditis patients, at first glance, may provide some suggestion of differentiation against ICI myocarditis patients. However, further examination of the pattern of LGE indicated that both conditions have a similar non-ischaemic LGE distribution (mostly subepicardial or mid-wall), which casts doubts over the ability of LGE to distinguish between ICI vs. non-ICI myocarditis.

Future work, including integrated scores for assessing structural heart diseases, may provide insights into a more comprehensive differentiation of ICI and non-ICI myocarditis [35]. Integrated scores, generated from multi-modality biomarkers, may enable the assessment of structural, functional, and electromechanical abnormalities in heart diseases [35]. In addition to direct myocardial inflammation, autonomic nervous system dysregulation has also been shown to play an important part in immune-mediated cardiac injury, as demonstrated in systemic autoimmune diseases such as systemic lupus erythematosus [36]. Future studies which assess the changes in the autonomic nervous system may uncover new avenues for clinical risk stratification and therapies in ICI myocarditis [36].

### 4.3. Limitations and Future Directions

This single-centred retrospective study was based on a relatively small sample size, rendering it susceptible to sampling bias and prohibiting meaningful power calculations. Larger studies are required to further test the ability of CMR to distinguish between ICI myocarditis and non-ICI myocarditis. The lack of quantitative LGE analysis meant that focal fibrosis burden was not part of the study analysis. However, quantitative LGE is not currently a part of routine clinical reporting in our centre for the assessment of acute myocarditis, but may offer insights in future research studies. The non-ICI myocarditis patient cohort was non-selective by aetiology and may contain a mixed effect from patients with different underlying causes of myocarditis. Further studies comparing different aetiologies of non-ICI myocarditis with ICI myocarditis may provide additional stratification of the results. Most patients with ICI myocarditis were already treated with immunosuppressive therapy before the CMR scans were performed. This meant that the degree of myocardial inflammation may be significantly less than at the original onset of myocarditis. Most of the non-ICI myocarditis patients did not receive immunosuppression. It may be possible that had the CMR been performed at the onset of ICI myocarditis and before immunosuppressive therapy was commenced, a higher level of parametric mapping results may have been detected. Further work is needed to assess the effect of early CMR in ICI myocarditis patients on the results, as compared to patients with non-ICI myocarditis. Synthetic ECV was used due to the lack of routine blood sample availability with the CMR scans. Although less validated in terms of normal thresholds and reproducibility, recent evidence does suggest that synthetic ECV correlates extremely well with laboratory ECV [37], which supports the validity of the study findings. The image analysis was not blinded to the ICI vs. non-ICI status of the patients, which may introduce subconscious bias. However, the analysis was performed using standardised and accepted methods, which may reduce this bias. Patients with ICI and non-ICI myocarditis were not age-matched, which reflects our clinical experience in that ICI myocarditis patients tend to be older. However, it is important to acknowledge that age can have an effect on the parametric mapping values, and future studies are required to address the individual and population-based impact of age on these conditions. A single mid-ventricular slice parametric map may under-detect focal disease, and future work involving mapping with whole-heart coverage would provide more insights. Finally, no patients in the study underwent myocardial biopsy, which is not commonly performed in our centre for the assessment of non-life-threatening myocarditis. It was therefore difficult to correlate the CMR findings with tissue structural changes in myocarditis.

## 5. Conclusions

In this single-centred retrospective cohort, the findings suggest that quantitative parametric mapping methods by CMR may not differentiate between ICI vs. non-ICI myocarditis. Further work is needed to assess the value of CMR for diagnosing standalone ICI myocarditis.

## Figures and Tables

**Table 1:** Clinical characteristics of immune checkpoint inhibitor (ICI) vs. non-ICI myocarditis.

	ICI Myocarditis (*n* = 26)	Non-ICI Myocarditis (*n* = 28)	*p*-Value
Age, years	75 (71–78)	39 (30–64)	<0.001
Male	15 (58)	18 (64)	0.781
Body mass index, kg/m^2^	22 (22–26)	25 (22–27)	0.135
Body surface area, m^2^	1.8 ± 0.3	2.0 ± 0.2	0.081
Cardiac symptoms			
Chest pain	1 (4)	19 (68)	<0.001
Dyspnoea	4 (15)	11 (39)	0.070
Palpitations	1 (4)	2 (7)	1.000
Syncope/Presyncope	0 (0)	3 (11)	0.237
Co-morbidities			
Diabetes mellitus	3 (12)	2 (7)	0.663
Hypertension	7 (27)	4 (14)	0.320
Hypercholesterolaemia	5 (19)	2 (7)	0.243
Chronic kidney disease	2 (8)	2 (7)	1.000
Atrial fibrillation	6 (23)	3 (11)	0.286
Ischaemic heart disease	5 (19)	1 (4)	0.095
COPD/Asthma	3 (12)	1 (4)	0.342
Stroke/TIA	1 (4)	0 (0)	0.481
Medications			
ACE-I/ARB/ARNI	16 (62)	8 (29)	0.027
Beta-blocker	19 (73)	11 (39)	0.016
MRA	8 (31)	1 (4)	0.010
SGLT-2 inhibitor	14 (54)	5 (18)	0.010
Loop diuretics	7 (27)	3 (11)	0.169
Anti-platelet drugs	7 (27)	4 (14)	0.320
Statins	5 (19)	4 (14)	0.724
Anticoagulation	4 (15)	3 (11)	0.699
Serum biomarkers			
Peak CRP, mg/L	113 (37–148) (*n* = 25)	47 (24–136) (*n* = 22)	0.371
Peak Hs-cTnT, ng/L	77 (51–169)	294 (57–1018) (*n* = 25)	0.039
Peak NT-pro BNP, ng/L	7838 (2412–13,116)	1631 (612–4147) (*n* = 11)	0.015

ACE-I: angiotensin converting enzyme inhibitor; ARB: angiotensin receptor blocker; ARNI: Angiotensin Receptor-Neprilysin Inhibitor; COPD: chronic obstructive airways disease; CRP: C-reactive protein; Hs-cTnT: high sensitivity cardiac troponin T; MRA: mineralocorticoid receptor antagonist; NT-pro BNP: N-terminal pro B-type natriuretic peptide; SGLT-2: Sodium-glucose co-transporter-2; TIA: transient ischaemic attack. Continuous data are expressed as mean ± SD or median (interquartile range). Categorical data were displayed as numbers (%).

**Table 2:** Characteristics of patients with immune checkpoint inhibitor (ICI) myocarditis.

	ICI Myocarditis (*n* = 26)
Primary malignancy site	
Lung	10 (38)
Skin	7 (27)
Tongue	3 (12)
Bladder	2 (8)
Thyroid	1 (4)
Breast	1 (4)
Cholangiocarcinoma	1 (4)
Renal	1 (4)
ICI agent	
Pembrolizumab	8 (31)
Ipilimumab/Nivolumab	5 (19)
Atezolizumab	3 (12)
Cemiplimab	3 (12)
Nivolumab alone	2 (8)
Durvalumab	2 (8)
Avelumab	1 (4)
Denosumab	1 (4)
Lenvatinib	1 (4)
Immunosuppressive therapy	
IV methylprednisolone	21 (81)
Oral prednisolone	24 (92)
Mycophenolate mofetil	15 (58)
Tacrolimus	5 (19)
Infliximab	5 (19)
Azathioprine	3 (12)
Tocilizumab	2 (8)
Methotrexate	1 (4)

IV: intravenous.

**Table 3:** CMR parameters in immune checkpoint inhibitor (ICI) vs. non-ICI myocarditis patients.

	ICI Myocarditis (*n* = 26)	Non-ICI Myocarditis (*n* = 28)	*p*-Value
CMR volumes and function			
LV EDVi, mL/m^2^	74 ± 20	84 ± 21	0.072
LV ESVi, mL/m^2^	33 ± 15	36 ± 13	0.325
LV SVi, mL/m^2^	42 ± 9	48 ± 10	0.022
LV EF, %	58 ± 11	58 ± 6	0.970
RV EDVi, mL/m^2^	69 (61–89)	74 (63–100)	0.229
RV ESVi, mL/m^2^	31 (23–37)	32 (22–45)	0.788
RV SVi, mL/m^2^	42 ± 12	47 ± 10	0.075
RV EF, %	57 ± 6	59 ±10	0.341
LV mass index, g/m^2^	52 (44–57)	58 (48–67)	0.051
LGE data			
LV LGE present	12 (52) (*n* = 23)	24 (89) (*n* = 27)	0.005
Subepicardial/mid-wall	0 (0)	11 (41)	<0.001
Mid-wall only	9 (39)	9 (33)	0.771
Subepicardial only	0 (0)	2 (7)	0.493
Transmural/Subendocardial	3 (13)	2 (7)	0.651
RV LGE present	0 (0)	0 (0)	-
Native myocardial T1 values (ms)	1041 ± 84 (*n* = 25)	1063 ± 60	0.281
Native myocardial T2 values (ms)	59 ± 6	59 ± 6 (27)	0.943
Synthetic ECV	0.32 ± 0.07 (*n* = 16)	0.31 ± 0.04 (21)	0.403

CMR: cardiovascular magnetic resonance; ECV: synthetic extracellular volume; EDVi: end-diastolic volume index; EF: ejection fraction; ESVi: end-systolic volume index; LGE: late gadolinium enhancement; LV: left ventricular; RV: right ventricular; SVi: stroke volume index. Continuous data were shown as mean ± SD or median (IQR). Categorical variables are shown as numbers (%).

## Data Availability

Patient clinical data in the study cannot be publicly shared but anonymised version can be provided on reasonable request to the corresponding author.
